# Occult mucin-producing cholangiocarcinoma in situ: a rare clinical case with difficult tumour staging

**DOI:** 10.1186/s40792-016-0283-x

**Published:** 2017-01-04

**Authors:** Muneyasu Kiriyama, Tomoki Ebata, Yukihiro Yokoyama, Tsuyoshi Igami, Gen Sugawara, Takashi Mizuno, Junpei Yamaguchi, Masato Nagino

**Affiliations:** Division of Surgical Oncology, Department of Surgery, Nagoya University Graduate School of Medicine, 65 Tsurumai-cho, Showa-ku, Nagoya, 466-8550 Japan

## Abstract

**Background:**

Mucin-producing cholangiocarcinoma (MPCC) is an uncommon tumour that is clinically characterized by mucin-hypersecretion. Because the initial symptoms of MPCC may be attributed to the viscus mucobilia, the primary tumour mass may potentially be unrecognizable. We report an interesting case of curatively resected occult MPCC in situ.

**Case presentation:**

A 70-year-old man was referred to our hospital with increased levels of biliary enzymes. Multidetector row computed tomography (MDCT) demonstrated a diffuse dilatation of the entire biliary system without evidence of tumour mass. Additionally, there were numerous variably sized cysts throughout the liver. The cyst of S4 was the largest, followed by that of S1, which connected with the right hepatic duct. Endoscopic retrograde cholangiography showed intrabiliary mucus, predominantly in the left hepatic duct, but failed to show a communication of both cysts with the bile duct. We clinically suspected that minute MPCC was present within the S1 cyst and performed left hepatectomy, caudate lobectomy, and resection of the extrahepatic bile duct. Macroscopically, papillary adenocarcinoma in situ was present in the S1 cyst, and a final diagnosis of MPCC originating from the bile duct of the caudate lobe was made.

**Conclusions:**

For MPCC, in practice, we should consider the possibility that this tumour can be occult. In this complicated setting, demonstrating the communication to the responsible dilated duct is a clue to the diagnosis. Multidirectional MDCT images succeeded in specifically demonstrating this communication, which is insensitive to the presence of excessive mucobilia.

## Background

Some cholangiocarcinomas secrete excessive mucus into the biliary system [1–4], causing dilatation of the entire biliary system and enlargement of the orifice at the papilla of Vater. These clinical features are uncommon in ordinary cholangiocarcinoma; therefore, this form of cholangiocarcinoma is clinically termed mucin-producing cholangiocarcinoma (MPCC) [1, 4, 5]. Associated symptoms are attributed to the viscous mucobilia rather than pathological obstruction of the bile duct. Therefore, the primary tumour may be potentially minute or unrecognizable if the tumour secretes excessive mucus [2, 3, 6, 7].

Here, we report a rare case with occult MPCC in situ that was resected with tumour-free margins. Lack of radiological evidence of a gross tumour and the presence of multiple liver cysts complicated the preoperative tumour diagnosis, thereby making surgical management difficult.

## Case presentation

**Fig. 1:**
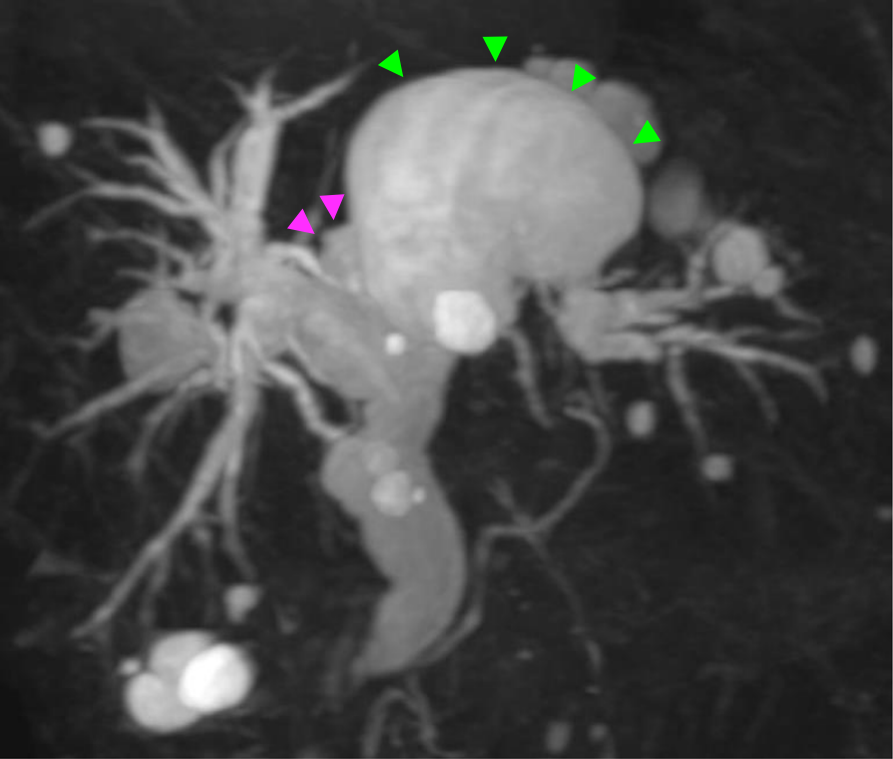
Magnetic resonance cholangiopancreatography. Multiple liver cysts, including the S1 cyst (*pink arrow head*) and the S4 cyst (*green arrow head*), were found. The whole bile duct was extensively dilated

A 70-year-old man had visited a local physician and had increased levels of biliary enzymes at medical check-up: serum aspartate aminotransferase (72 IU/l), alanine aminotransferase (212 IU/l), alkaline phosphatase (555 IU/l), gamma glutamyl transpeptidase (218 IU/l), and total bilirubin (0.4 mg/dL). He had undergone open cholecystectomy 23 years ago. Magnetic resonance cholangiopancreatography (MRCP) revealed a diffuse dilatation of the entire biliary system and bilateral multiple cysts in the liver (Fig. 1). He was referred to our hospital for further evaluation.

**Fig. 2:**
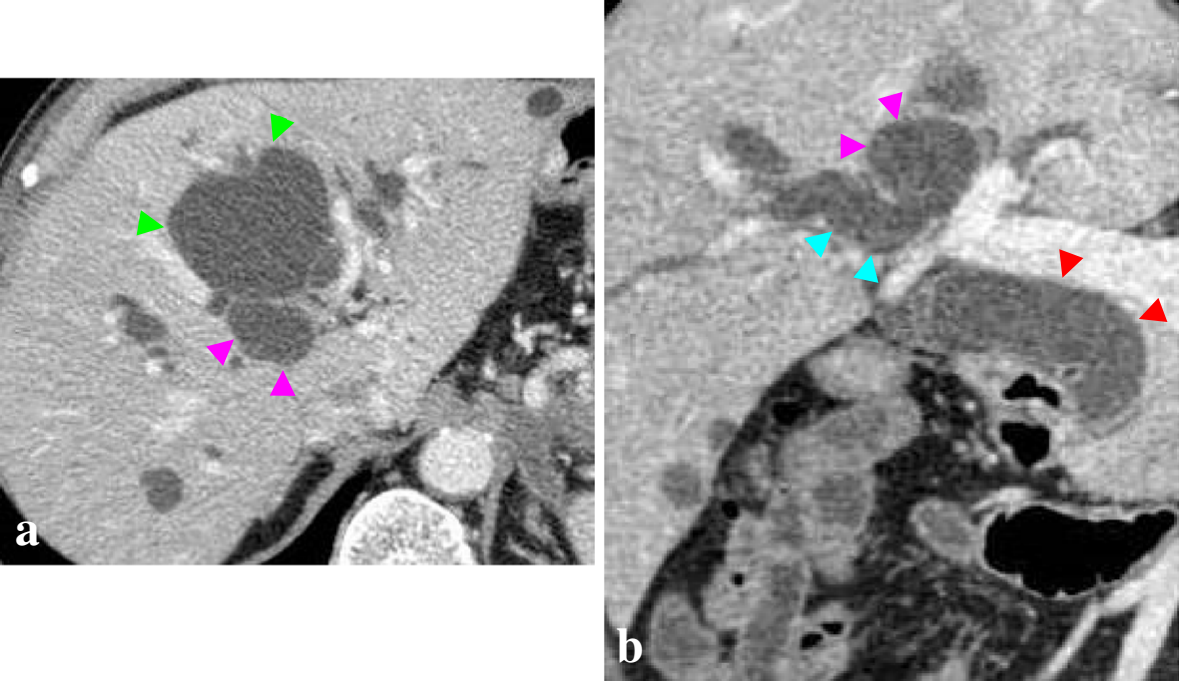
Multidetector row computed tomography. The S1 cyst (*pink arrow head*) connected with the right hepatic duct (*blue arrow head*). The extrahepatic bile ducts were diffusely dilated (*red arrow head*). The tumour mass, stricture, and wall thickness were not evident in either cyst or the entire bile duct. The *green arrow head* shows the S4 cyst. **a** Axial image. **b** Coronal image

**Fig. 3:**
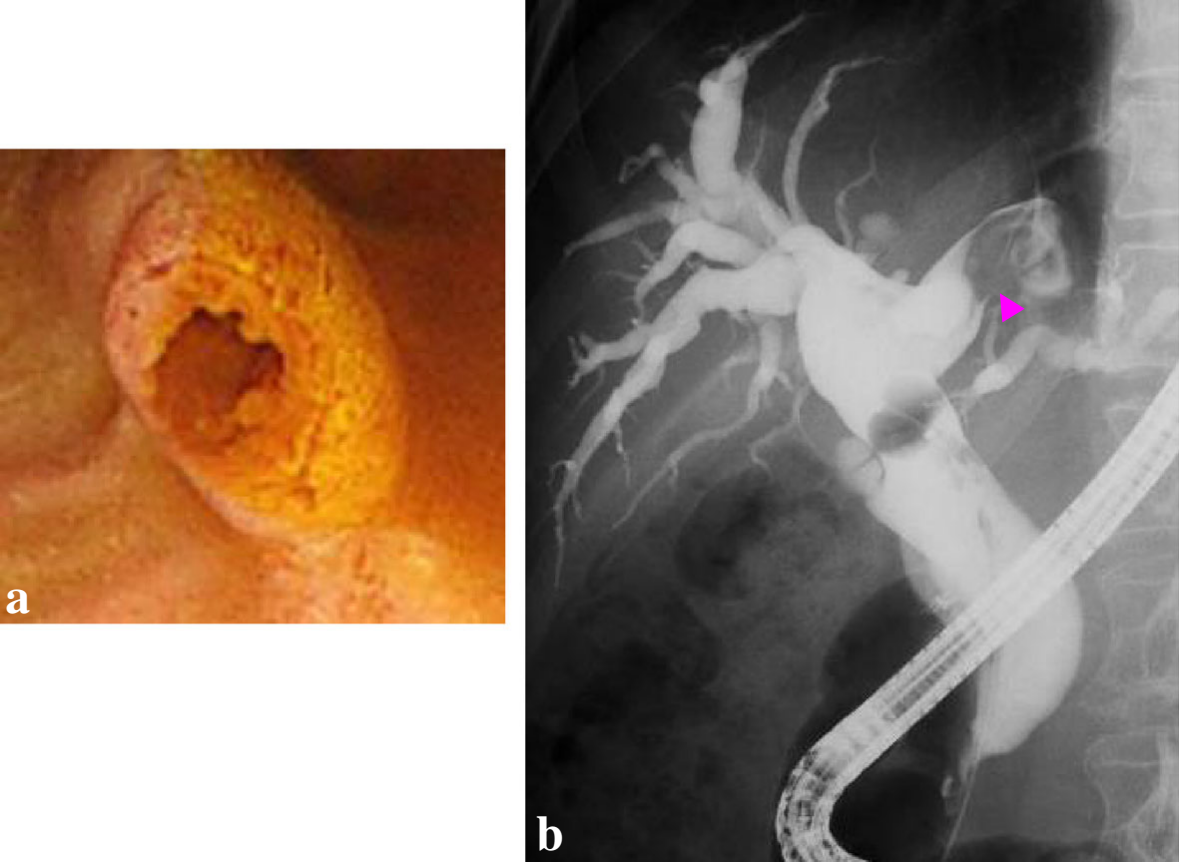
**a**. Duodenoscopy. The orifice of the Vater’s papilla was expanded by mucus. **b**. Endoscopic retrograde cholangiography. The entire bile duct was diffusely dilated. There was floating mucus in the left hepatic duct (*pink arrow head*)

Multidetector row computed tomography (MDCT) revealed extensive dilatation of the entire bile duct without evidence of tumour mass or stricture. Among the liver cysts, there were two major adjacent cysts in S1 (33.1 mm in size) and S4 (74.7 mm in size). The former was evidently connected with the right hepatic duct, whereas the latter was not (Fig. 2). Duodenoscopy revealed a dilated orifice of the Vater’s papilla with mucus, indicating the mucin-hypersecreting nature of the disease (Fig. 3a). Endoscopic retrograde cholangiography (ERC) demonstrated mobile ragged translucencies (i.e., intrabiliary mucus), predominantly in the left hepatic duct. ERC failed to demonstrate a communication between the cysts and the bile duct (Fig. 3b). The four biopsy samples taken from the right, left, hilar, and upper bile ducts were all negative for cancer.

The above radiological findings strongly suggested the presence of minute/occult MPCC. For the surgical management, we focused on two radiologic findings: first, the S1 cyst connected to the bile duct, suggesting a primary diseased site; second, mucus was found predominantly in the left hepatic duct, suggesting that the largest S4 cyst should also be resected. Therefore, we planned to perform left hepatectomy, caudate lobectomy, and extrahepatic bile duct resection with dissection of regional lymph nodes. During surgery, because the two cysts adhered to the middle hepatic vein, liver transection was carefully advanced to avoid rupture. We resected the two cysts en bloc without explosion. The operation time was 484 min, and blood loss was 913 ml. The patient’s postoperative course was uneventful, except for minor bile leakage, and he was discharged 16 days after the surgery.

**Fig. 4:**
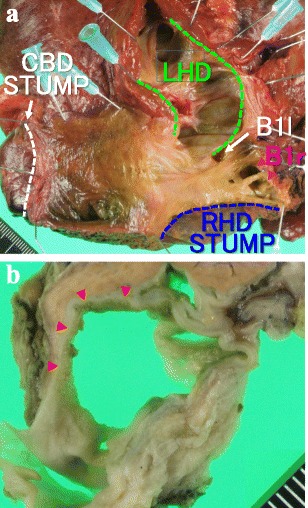
Macroscopic findings. **a**. The S1 cystic lesion connected with right hepatic duct via the two bile ducts of the right caudate lobe (*pink arrow head*). (*CBD* common bile duct, *LHD* left hepatic duct, *RHD* right hepatic duct, *B1l* confluence of the left caudate branch, *B1r*: confluence of the right caudate branch). **b**. Cut surface of the S1 cyst. Fine granular tumour spreads in the luminal surface (*red arrow head*)

**Fig. 5:**
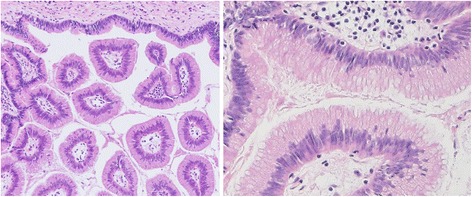
Histological findings. Papillary projection, which was formed of atypical epithelial cells with multilayering nuclei and moderately increased N/C ratio is present in the S1 cyst. Because of the marked papillary structural atypia, despite moderate cellular atypia, the diagnosis of papillary adenocarcinoma in situ was made

Macroscopically, the S1 cystic lesion, filled with mucus, connected with the right hepatic duct via the two bile ducts of the right caudate lobe (B1r), the luminal surface of which showed a fine granular pattern (Fig. 4a, b). Microscopically, papillary projection into the lumen, formed of atypical epithelial cells with multilayering nuclei, was found, indicating papillary adenocarcinoma in situ (Fig. 5). A papillary to flat lesion with variable degrees of cellular atypia spread to the B1r. All regional lymph nodes were negative for cancer. According to the American Joint Committee on Cancer [8], the tumour was labeled as stage 0 (TisN0M0) disease. The S4 cyst was a simple cyst without neoplastic epithelium. All margins were tumour-free. Definitive diagnosis of MPCC in situ developing from the B1r was made. The patient remains well without tumour recurrence 3 years and 9 months after the surgery.

## Discussion

MPCC was first reported by Chou et al. [9] in 1976; thereafter, the disease concept has been described by Nagino et al. [1]. MPCC is not a pathological but a clinical disease entity characterized by mucin-hypersecretion, although its extent has not been standardized. Several investigators verified that MPCC histologically exhibits an intraductal papillary proliferation along with delicate fibrovascular cores; heterogenous atypia of the tumour cells; and frequent flat intraepithelial lesion around the primary tumour [10, 11]. These features suggest that MPCC is an overlapping disease category with intraductal papillary neoplasm of the bile duct (IPNB) [10, 12–14]. In this report, we used the term MPCC in the historical sense for the detection of the disease. Alternative terminology of IPNB was also possible in this patient, although the definition of IPNB has not been consistent.

Yang et al. [6] showed that the extent of mucin-secretion bears no relationship to the atypia of the tumour cells; some mucin-producing tumours are pathologically diagnosed as adenoma. Onoe et al. [15] also reported that pathologically malignant potentials representing size, tumour depth, or nodal status were comparable between papillary cholangiocarcinomas with mucin-secretion and those without. These observations fail to confirm specific pathological features and, in turn, suggest a morphological variation in MPCC. In fact, Yeh et al. [16] retrospectively analyzed cholangiographic findings in 13 patients with histologically proven MPCC, of which 8 patients had a finding of mucobilia without gross tumour. Surprisingly, one-third of patients with MPCC had occult (radiologically invisible) tumours. Overall, the following three clinical lessons should be considered: first, mucobilia is a sentinel sign of MPCC; second, MPCC exhibits a variety of morphologies; and third, gross neoplasia is unrecognized more frequently than expected. Thus, we should revise our diagnostic strategy to be applicable to patients with a sole sign of mucobilia (occult MPCC).

MPCC exhibits two representative morphologies: ductectatic type and cystic type. The former is defined as a papillary tumour in the dilated intrahepatic bile duct branch that shows disproportional enlargement, and the latter is defined as a papillary tumour within a large liver cyst that communicates with the biliary system [4]. Notably, saturating mucus usually fills not only the diseased cyst but also the communication; therefore, conventional cholangiography has limited value unless specific preparations are performed [17]. In contrast, the presence of mucin never impairs MDCT images, allowing dilated bile ducts to be easily evaluated by multidirectional reconstructions and by paging view on electronic medical records systems. Therefore, MDCT is an important alternative to cholangiography, particularly in occult MPCC.

In the present case, we hypothesized that the occult MPCC was associated with any of the liver cysts (a variation of cystic type MPCC). As the communication between the S1 cyst and the hepatic duct was clearly demonstrated on CT images, the S1 cyst was specified as the target region. Additionally, biopsy samples from the bile duct were all negative for cancer, indicating that the tumour was confined to the cyst and the communication. Based on this limited information, we planned left hepatectomy, caudate lobectomy, and extrahepatic bile duct resection to maximize the chance of R0 resection.

The clinical impact of the remnant carcinoma in situ at the ductal remains a matter of debate; however, our recent study clearly demonstrates the growing nature of the foci, the risk of local disease relapse, and a deteriorating impact on postoperative survival [18]. These biological behaviours, however, are observed only in patients with early stage cholangiocarcinoma. In another words, ductal margin status is a sole prognostic factor in an early stage of the disease (occult MPCC) [19]. The diagnostic utility of the peroral cholangioscopy (POCS) tool has been reported [5, 20, 21]. POCS provides an accurate diagnostic yield regarding intraepithelial neoplasia in the large bile duct. In this study, we unfortunately did not perform POCS, mainly due to the endoscopists’ preference.

## Conclusions

Occult MPCC is uncommon and complicated. MDCT is a leading diagnostic modality in which a communication between the cystic lesion and the biliary system is the key finding to specify the disease site. Although an optimal approach for occult MPCC has not been fully addressed, this case report provides a simple MDCT-based diagnostic approach and surgical management for this challenging scenario.
